# Psychological distress is involved in CRCI in breast cancer survivors via mediating cytokine levels

**DOI:** 10.1002/cam4.5847

**Published:** 2023-03-25

**Authors:** Lulian Pang, Wen Li, Senbang Yao, Yanyan Jing, Xiangxiang Yin, Huaidong Cheng

**Affiliations:** ^1^ Department of Oncology the Second Affiliated Hospital of Anhui Medical University Hefei Anhui China; ^2^ The Third School of Clinical Medicine, Southern Medical University Guangzhou China; ^3^ Department of Oncology Shenzhen Hospital of Southern Medical University Shenzhen China

**Keywords:** breast cancer, cancer‐related cognitive impairment, cytokines, mediate, psychological distress

## Abstract

**Background:**

Cancer‐related cognitive impairment (CRCI) is a frequent consequence in breast cancer survivors after chemotherapy and lowers their quality of life (QOL). Psychological distress is frequently experienced by breast cancer survivors. There are currently few studies investigating the role of psychological distress in the genesis of CRCI.

**Methods:**

In total, 122 breast cancer survivors after standard chemotherapy within a year were recruited and assessed using the Psychological Distress Thermometer (DT). Sixty breast cancer survivors had non‐psychological distress (NPD group) and sixty‐two breast cancer survivors with psychological distress (PD group). The scores of the Mini‐Mental State Examination (MMSE), prospective and retrospective memory (PM and RM) Questionnaire (PRMQ), and Functional Assessment of Cancer Therapy‐General (FACT‐G) and the levels of cytokines including interleukin‐1 beta (IL‐1β), tumor necrosis factor‐alpha (TNF‐α), and interleukin‐4 (IL‐4) were compared between the two groups. Using PROCESS, we investigated whether psychological distress predicted cognitive function based on MMSE through IL‐1β, TNF‐α, and IL‐4.

**Results:**

The PD group had higher scores on RM, PM, and FACT‐G and lower scores on MMSE than the NPD group (*t* = −11.357, *t* = −10.720, *t* = −15.419, *t* = 10.162, respectively; *p* < 0.05). Meanwhile, a higher level of IL‐1β, TNF‐α, and IL‐4 was observed in the PD group than in the NPD group (*t* = −3.961, *t* = −3.396, *t* = −3.269, respectively; *p* < 0.05). The link between psychological distress and cognitive function as measured by the MMSE was also mediated by IL‐1β, TNF‐α, and IL‐4 (effect size: 26%, 25%, and 24%).

**Conclusion:**

Breast cancer patients with psychological distress displayed poor cognitive function, poor memory, and inferior quality of life, which was accompanied by higher cytokine levels of IL‐1β, TNF‐α, and IL‐4. This study demonstrated IL‐1β, TNF‐α, and IL‐4 as potential pathways to CRCI in response to ongoing psychological distress, which provided evidence for the involvement of psychological distress in CRCI in breast cancer survivors.

## Graphical abstract

Psychological distress and cognitive function in breast cancer patients were assessed by questionnaires and peripheral blood cytokine levels were measured. Psychological distress was involved in chemotherapy‐related cognitive impairment (CRCI) by increasing IL‐1β, TNF‐α, and IL‐4 levels.

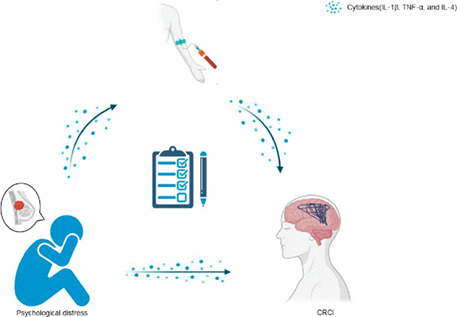

## 1 INTRODUCTION

Since 2020, breast cancer has become the most common cancer in the world, ranking fifth in all cancers in terms of mortality.^1^ From 2015 to 2020, the number of new cases of breast cancer in China reached 112,000, and the related deaths already reached 47,000, accounting for 18.41 percent of the total global cases and 17.11% of the global breast cancer deaths.^2^ Despite this, from 1989 to 2017, related death rates decreased by about 40%.^3^ Chemotherapy is an important therapeutic agent for improving the overall survival of breast cancer patients. Due to the updating of therapeutic drugs, the 15‐year survival rate of early breast cancer has reached 77.6%.^4^ Therefore, scientists are becoming more aware of the side effects of these treatments. In addition to adverse reactions such as nausea, vomiting, hair loss, and bone marrow suppression, cancer‐related cognitive impairment (CRCI) also harms breast cancer survivors.^5^, ^6^

CRCI is a common complication in breast cancer patients and refers to cognitive dysfunction including short‐term memory problems, attention difficulties, executive function problems, and processing speed problems.^7^, ^8^ Related studies showed that CRCI not only decreases the quality of life but also continues to impact the work reintegration of breast cancer patients after treatment.^9^ It has been shown that cognitive impairments may persist in breast cancer survivors for 5–10 years after treatment.^10^ Mechanisms driving cognitive dysfunction after chemotherapy may include oxidative stress and inflammation, decreased neurogenesis, changes in neuronal dendrites and axons, and microglial apoptosis.^11^, ^12^, ^13^ A systematic review by Tyagi et al^14^ presented that IL‐6, IL‐1β, and TNF‐α were associated with cognitive dysfunction in post‐chemotherapy breast cancer patients.

Although CRCI was reported as mainly occurring in breast cancer patients after a variety of treatments, including chemotherapy, it will also be detected before cancer treatment.^15^, ^16^ In a study by Dijkshoorn et al,^17^ a quarter of breast cancer patients have cognitive impairment before receiving chemotherapy. Researchers have found that newly diagnosed breast cancer patients without any treatment (including surgery), had significantly lower than that of matched cancer‐free participants in cognitive function.^18^ Relatively few studies pay attention to the psychological factors behind CRCI. Recent research demonstrated that lower self‐esteem is linked to cognitive concerns in individuals.^19^ Psychological variables were found to be associated with CRCI among breast cancer patients in a review.^20^ Cancer is a major life‐changing event that causes a considerable amount of psychological distress. Psychological distress is a complicated emotional state that may be caused by difficult‐to‐solve practical issues, communication problems, emotional problems, faith/religious problems, as well as a compromised physical condition brought on by a tumor and its treatment. According to Areklett et al,^21^ psychological distress, including anxiety and depression, raised the incidence of CRCI in cervical cancer survivors by 13% and 16%, respectively. Therefore, it is important to continue researching if psychological distress contributes to the development of CRCI in breast cancer survivors and its potential path of participation.

The pathological changes caused by chemotherapy often include inflammation in breast cancer patients with cognitive dysfunction.^22^, ^23^ However, cancer patients before treatment had higher levels of inflammatory cytokines such as IL‐2, IL‐4, IL‐6, IL‐8, and IL‐10 than healthy controls and performed worse on cognitive tests.^24^ In addition, there is a causal link between circulating cytokine concentrations and Alzheimer's disease, with IL‐4 being associated with higher fluid intelligence.^25^ A review by Yu et al^26^ demonstrated that cytokines such as IL‐1Ra, IL‐6, and TNF‐α increase the permeability and dysfunction of the blood–brain barrier (BBB) and promote the occurrence of depression in breast cancer patients. The mechanism of inflammatory markers in CRCI remains unclear, and the correlation between inflammatory factors and negative emotions in cancer patients is rarely studied.

In brief, psychological health is easily threatened by life‐limiting diseases like cancer. Breast cancer patients experienced varying degrees of psychological distress and cognitive impairment before and after treatment. At present, more researchers focus on the physiological mechanism of CRCI, but few studies pay attention to the psychological factors. This study further demonstrated whether psychological distress could affect cognitive function by influencing the level of IL‐1β, TNF‐α, and IL‐4, which provides evidence for paying increasing attention to the mental health of breast cancer patients and early implementation of psychological intervention.

## 2 METHODS

### 2.1 Participants

In this study, which originated from 2019 to 2020, 122 patients with early‐stage breast cancer from the Second Affiliated Hospital of Anhui Medical University were enrolled within a year of completing chemotherapy based on taxanes and anthracyclines four to six times. The study's inclusion criteria were as follows: (1) patients with pathologically diagnosed breast cancer who have completed chemotherapy based on taxanes and anthracyclines 4–6 times; (2) Adequate bone marrow and organ reserve: neutrophils ≥2.0 × 109, platelet ≥100 × 109, hemoglobin ≥90 g/L; Urinary creatinine <80 μmol/L; Aspartate aminotransferase, alanine aminotransferase ≤3 times the normal value; Bilirubin level ≤1.5 times the normal value; (3) With primary school education or above, being able to complete the questionnaires independently. The exclusion criteria: (1) Complicated with other serious diseases, such as serious infections and autoimmune diseases; (2) Previous history of mental and neurodegenerative diseases, such as depression, schizophrenia, dementia, and Alzheimer's disease (3) Patients with breast cancer brain metastasis or cognitive impairment due to neurological histories such as stroke via imaging examination and history‐taking; (4) Have a language barrier or are unable to express themselves clearly.

### 2.2 Assessments

#### 2.2.1 Distress

The psychological Distress Thermometer (DT)^27^ is a self‐assessment of the participants' psychological distress levels for the past 1 week (including today), including practical, communicated, emotional, spiritual/religious, and physical problems. Psychological distress is defined as a score of 4 or more on a scale ranging from 0 to 10.^28^ The higher scores represent the more severe psychological distress.

#### 2.2.2 Cognitive

The Mini‐Mental State Examination (MMSE)^29^ was a cognitive screening test used to measure the overall cognitive function, including temporal and spatial orientation, memory, attention and computation, recall, language, and executive function, with total scores from 1 to 30, with higher scores indicating better cognitive functioning. MMSE scores of 26 or less are considered cognitively impaired, whereas scores of 27–30 are considered normal.

#### 2.2.3 Memory

The prospective and retrospective memory questionnaire (PRMQ)^30^ is used to assess daily memory loss. There are 16 self‐reported items, and each item is divided into four levels. A score of 1–4 representing “never”, “sometimes”, “often” and “always” was used to test prospective and retrospective memory, respectively. Those with higher scores have a more severe impairment of memory.

#### 2.2.4 Quality of life

Patients are assessed on four dimensions of quality of life using the Functional Assessment of Cancer Therapy‐General (FACT‐G) questionnaire.^31^ It consists of 27 items and 9 additional questions covering physical status, social/family status, emotional status, and functional status. Physical and emotional problems were rated on five tiers, with 0–4 scores representing “not at all (=0)”, “a little (=1)”, “somewhat (=2)”, “quite (=3)”, and “very much (=4)”, social/family status and functional status were scored oppositely, higher scores indicating poorer quality of life.

### 2.3 Cytokine measurements

Two milliliters of venous blood were collected from each patient and placed in ethylenediaminetetraacetic acid (EDTA) tubes. The plasma was separated from all samples by centrifugation at 3000 RCF for 10 min. We reserved at least 600 μL of each sample for testing and stored them aseptically at −80°C. The cryopreserved specimens were tested for cytokine levels by ELISA at Shanghai Tianhao Biological company. The catalog numbers for the ELISA kit of IL‐1β, TNF‐α, and IL‐4 were ml058059, ml077385, and ml058093 and their specifications were 96T.

### 2.4 Design and procedure

**FIGURE 1:**
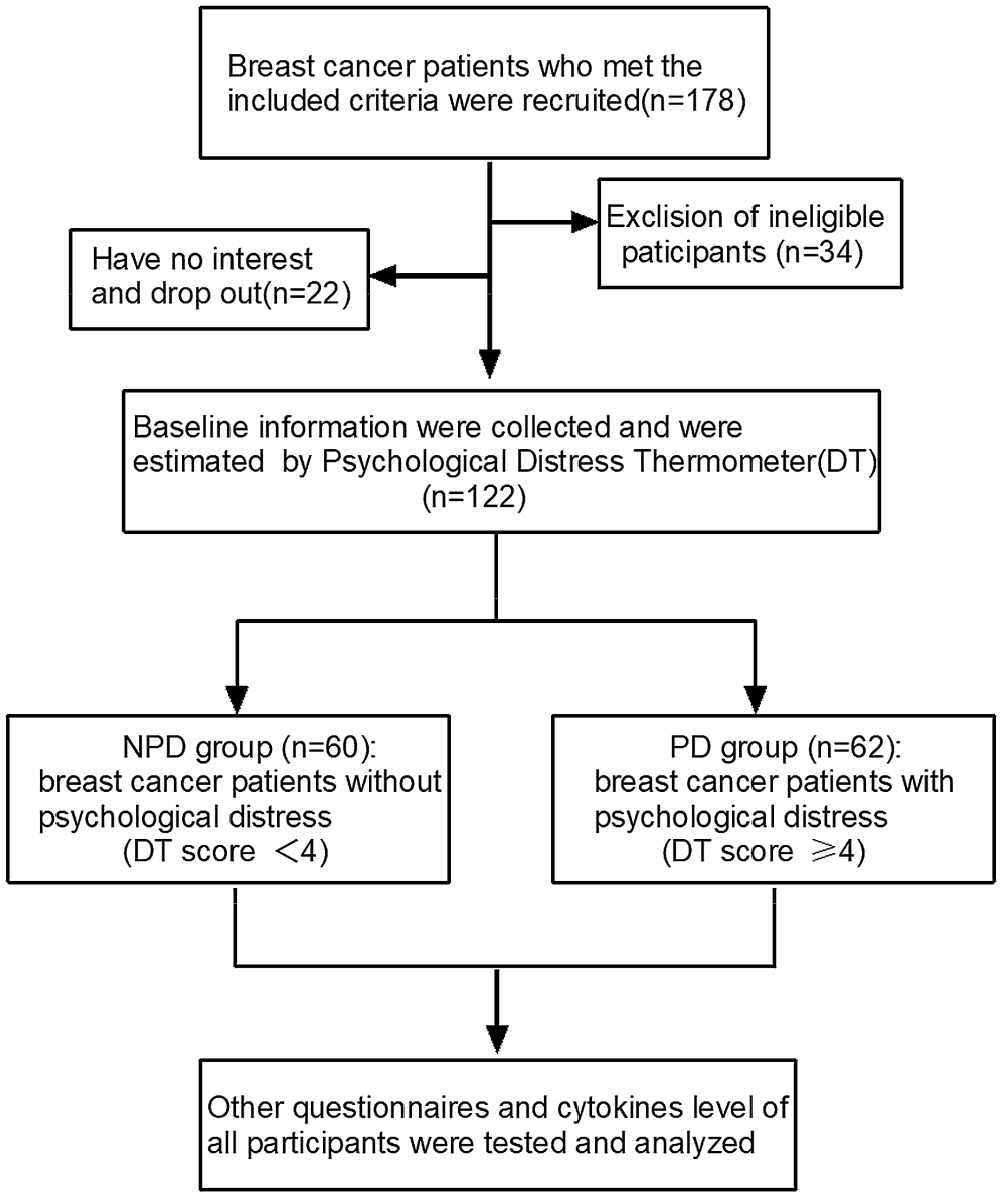
Flowchart of study design.

In this study, patients with early‐stage breast cancer were divided into two groups by DT. Breast cancer patients whose DT scores of 4 or more were assigned to the psychological distress (PD) group, whose DT scores of less than 4 were in the non‐psychological distress (NPD) group, then the neuropsychological tests and self‐reported questionnaires were evaluated, and cytokines levels including IL‐1β, TNF‐α, IL‐4 were detected in the two groups, as showed in Figure 1.

### 2.5 Statistical analysis

A power analysis was conducted using G*Power 3.1.^32^ In a multiple regression model with three predictors (effect size = 0.15, power level = 0.8, *p* = 0.05), a sample size of at least 55 participants was required to detect a moderate effect. Statistical Package for Social Sciences Statistics (Version 25) was used for descriptive analyses. Age, education, and Karnofsky performance status (KPS) scores were analyzed using an independent sample *t* test. The results including tumor stage, tumor size, pathological type, and HER‐2 expression were displayed by the Pearson Chi‐Square test except the surgical method was analyzed using Fisher's exact test. An independent sample *t* test was used to measure the differences between the NPD group and PD group in questionnaire scores and cytokines levels. All correlations were analyzed using linear correlation analysis. A *p* value of 0.05 was set when a significant result was found in all two‐tailed tests.

Using PROCESS macro (version 3.3) model 4 of SPSS,^33^ we tested the mediation model; psychological distress was taken as a predictor, IL‐1β, TNF‐α, and IL‐4 as mediators, and cognitive function (MMSE) as the outcome variable. PROCESS Model 4 examined the effect of psychological distress on IL‐1β, TNF‐α, and IL‐4 (A path), IL‐1β, TNF‐α, and IL‐4 on cognitive function (B path), the aggregate effect of the psychological distress on cognitive function (C path), and the direct effect of the psychological distress on the cognitive function in this model (C′ path). An indirect effect is considered significant when 0 does not appear in the 95% confidence interval (CI), indicating that CI is uniformly positive or negative. A mediation analysis was performed on IL‐1β, TNF‐α, and IL‐4, respectively.

## 3 RESULTS

### 3.1 Baseline data and clinical information

By DT, 122 breast cancer patients were divided into two groups: NPD (*n* = 60) and PD (*n* = 62). There were no significant differences in age (49.48 ± 7.00/51.37 ± 6.63), education level (9.12 ± 2.28/8.58 ± 1.97), and KPS score (88.00 ± 4.03/87.58 ± 4.32) between NPD group and PD group (*t* = −1.528, *t* = 1.388, *t* = 0.555, respectively; *p* > 0.05), were presented as mean ± SD, and there were no significant differences in tumor stage, tumor size, surgical method, pathological type and HER‐2 expression between the two groups (χ^2^ = 0.697, χ^2^ = 0.889, χ^2^ = 0.990, χ^2^ = 1.656, χ^2^ = 1.055, respectively; *p* > 0.05). Table 1 shows the results.

**TABLE 1:** The demographic characteristics and clinical information of the patients.

Variables	NPD (*n* = 60)			PD (*n* = 62)			χ^2^/*t*	*p*
	*N* (%)	Mean	SD	*N* (%)	Mean	SD		
Age		49.48	7.00		51.37	6.63	−1.528	0.129
Education		9.12	2.28		8.58	1.97	1.388	0.168
KPS		88.00	4.03		87.58	4.32	0.555	0.580
Tumor stage								
I	8 (13.3)			6 (9.7)			0.697	0.706
II	27 (45)			32 (51.6)				
III	25 (41.7)			24 (38.7)				
Tumor size								
< cm	15 (25.0)			19 (30.6)			0.889	0.641
2–5 cm	37 (61.7)			33 (53.2)				
>5 cm	8 (13.3)			10 (16.1)				
Surgery								
Mastectomy	50 (83.3)			52 (83.8)			0.990	0.719
Lumpectomy	8 (13.3)			5 (8.1)				
No surgery	2 (3.3)			3 (8.1)				
Pathology								
Infiltrative	36 (60.0)			30 (48.4)			1.656	0.209
Invasive ductal	24 (40.0)			32 (51.6)				
HER‐2								
HER‐2 (+)	47 (78.3)			53 (85.5)			1.055	0.352
HER‐2 (−)	13 (21.7)			9 (14.5)				

Abbreviation: KPS, Karnofsky performance status.

### 3.2 Comparison of questionnaires scores between the NPD group and PD group

**FIGURE 2:**
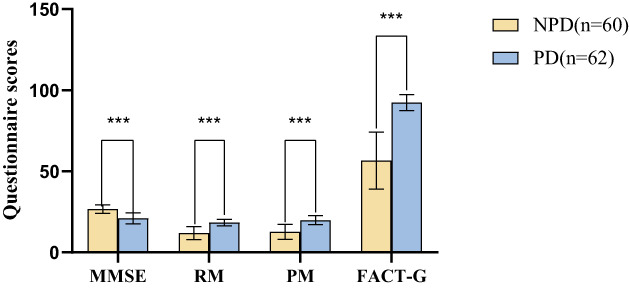
Comparison of MMSE, RM, PM, and QOL between NPD (*n* = 60) and PD (*n* = 62), the scores were expressed as mean ± standard deviation (SD), and the difference was measured using an independent sample *t* test (*p* < 0.05). Significant differences in MMSE, RM, PM, and QOL were observed between NPD (*n* = 60) and PD (*n* = 62).

MMSE (26.63 ± 2.63/21.08 ± 3.35), RM (11.95 ± 3.97/18.39 ± 2.01), PM (12.67 ± 4.59/19.97 ± 2.73), and FACT‐G (56.63 ± 17.57/92.40 ± 4.91) between NPD group and PD group were significantly different (*t* = 10.162, *t* = −11.357, *t* = −10.720, *t* = −15.419, respectively; *p* < 0.05). According to Table 2 and Figure 2.

**TABLE 2:** Comparison of MMSE, PM, RM, FACT‐Cog, and FACT‐G scores between the NPD group and PD group.

Items	NPD (*n* = 60)	PD (*n* = 62)	*t*	*p*
MMSE	26.63 ± 2.63	21.08 ± 3.35	10.162	<0.01
RM	11.95 ± 3.97	18.39 ± 2.01	−11.357	<0.01
PM	12.67 ± 4.59	19.97 ± 2.73	−10.720	<0.01
FACT‐G	56.63 ± 17.57	92.40 ± 4.91	−15.419	<0.01

Abbreviations: NPD, non‐psychological distress; PD, psychological distress.

### 3.3 Comparison of cytokines levels between the NPD group and PD group

**FIGURE 3:**
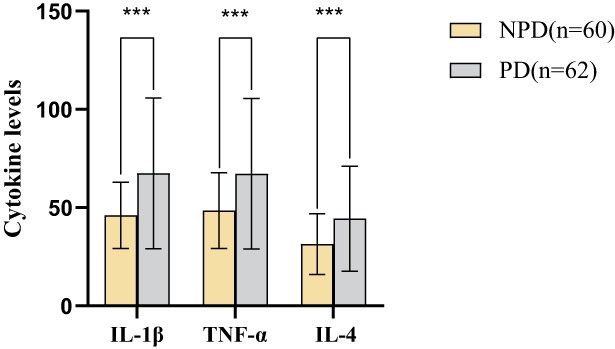
Histogram of comparison on cytokines level including IL‐1β, TNF‐α, and IL‐4 between NPD (*n* = 60) and PD (*n* = 62). All samples were centrifuged for 10 min at speeds of 2000 RCF, the plasma of the blood sample was separated by centrifugation, and the cytokine level of all samples was determined by ELISA, the unit of value is pg/mL. All data were expressed as mean ± SD, and the difference was measured using an independent sample *t* test (*p* < 0.05). The cytokine levels of IL‐1β, TNF‐α, and IL‐4 were significantly different between NPD (*n* = 60) and PD (*n* = 62).

The levels of the cytokines IL‐1β (46.06 ± 16.86/67.45 ± 38.38), TNF‐α (48.52 ± 19.34/67.26 ± 38.27), and IL‐4 (31.42 ± 15.54/44.38 ± 26.64) in NPD group were lower compared with PD group (*t* = −3.961, *t* = −3.396, *t* = −3.269, respectively; *p* < 0.05), as shown in Table 3 and Figure 3.

**TABLE 3:** Comparison of IL‐1β, TNF‐α, and IL‐4 levels between the NPD group and PD group.

Items	NPD (*n* = 60)	PD (*n* = 62)	*t*	*p*
IL‐1β	46.06 ± 16.86	67.45 ± 38.38	−3.961	<0.01
TNF‐α	48.52 ± 19.34	67.26 ± 38.27	−3.396	<0.01
IL‐4	31.42 ± 15.54	44.38 ± 26.64	−3.269	<0.01

Abbreviations: NPD, non‐psychological distress; PD, psychological distress.

### 3.4 Mediation analyses

**FIGURE 4:**
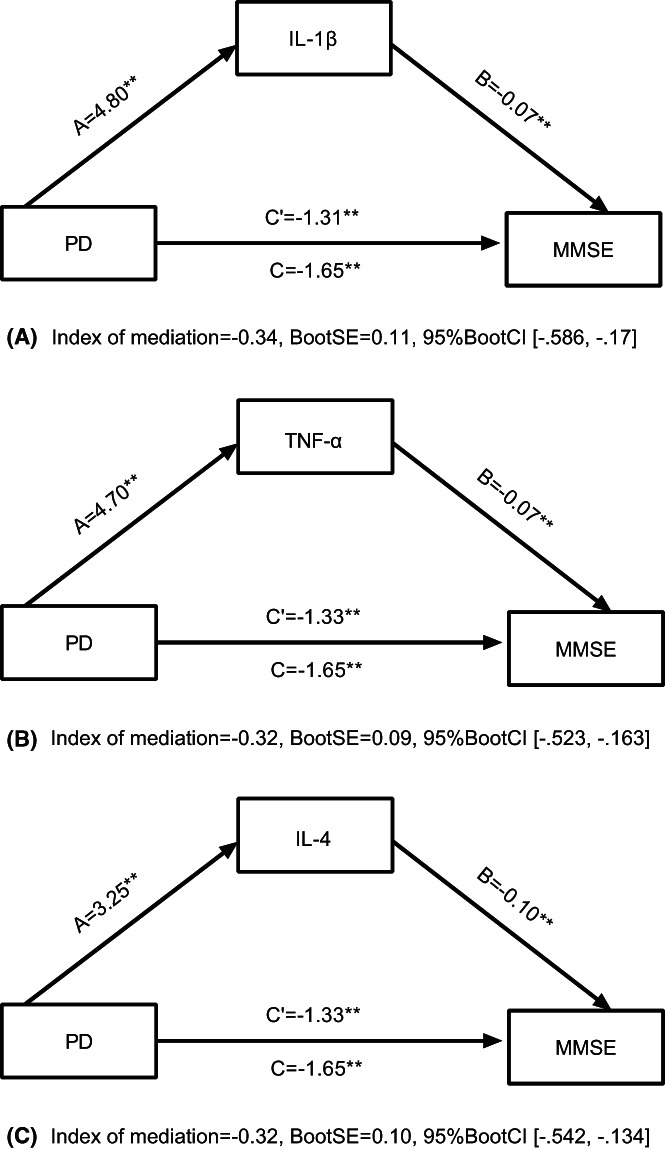
The partial mediation of psychological distress (PD) via IL‐1β, TNF‐α, and IL‐4 in predicting MMSE, as shown in figures A, B, C. Figure depicts the mediation model tested using Model 4 of Hayes'PROCESS in predicting cognitive function based on the Mini‐Mental State Examination (MMSE). *p < 0.05; **p < 0.01, (two‐tailed).

According to the mediation model, psychological distress had direct and indirect effects on cognitive function through cytokines. The results of mediation analyze for IL‐1β, TNF‐α, and IL‐4 were described in Table 4 and Figure 4. In all mediation models, the A path on behalf of the relationship between psychological distress and cytokine levels (IL‐1β, TNF‐α, and IL‐4) was significant and positive (=4.80, =4.70, =3.25, respectively; <0.05). The C path, representing the relationship between psychological distress and cognitive function, was significant and negative (=−0.07, =−0.07, =−0.10, respectively; <0.05), manifesting that psychological distress predicted cognitive function.

**TABLE 4:** Tests of mediation of PD by IL‐1β, TNF‐α, and IL‐4 in predicting MMSE.

Cytokine levels	Model summary	Effect of PD on cytokine levels (A path)	Effect of cytokine levels on MMSE (B path)	Total effect (C path)	Direct effect (C′ path)	Indirect effect (AB path)	95% LLCI	95% ULCI	Effect size (AB/C′)
IL‐1β	*R* = 0.24	4.80**	−0.07**	−1.65**	−1.31**	−0.34	−0.59	−0.17	0.26
	*R* ^2^ = 0.58								
	*F* = 7.33								
TNF‐α	*R* = 0.23	4.70**	−0.07**	−1.65**	−1.33**	−0.32	−0.52	−0.16	0.25
	*R* ^2^ = 0.05								
	*F* = 6.93								
IL‐4	*R* = 0.23	3.25*	−0.10**	−1.65**	−1.33**	−0.32	−0.54	−0.13	0.24
	*R* ^2^ = 0.05								
	*F* = 6.44								

^**^ *p*‐value at 0.01 level, the correlation was significant

* *p*‐value at 0.05 level, the correlation was significant.

The indirect effect of psychological distress via IL‐1β, TNF‐α, and IL‐4 on the MMSE was significant and negative (=−0.34, =−0.32, =−0.32, respectively; 95% CI [−0.59 to −0.17], [−0.52 to −0.16], [−0.54 to −0.13]). The direct effect of psychological distress on cognitive function (=−1.31, =−1.33, =−1.33, respectively; <0.05) in the three mediation models was decreased and significant compared with the total effect (=−1.65; <0.05). These results indicated the partial mediation of psychological distress via IL‐1β, TNF‐α, and IL‐4, in which psychological distress both directly predicted cognitive function as well as indirectly. The indirect effect size of psychological distress on cognitive function through IL‐1β, TNF‐α, and IL‐4 were 26%, 25%, and 24%, respectively.

## 4 DISCUSSION

Our study showed the mediating role of cytokines in the relationship between psychological distress and CRCI among breast cancer survivors. The present results suggest that breast cancer survivors with PD have worse cognitive functioning based on a cognitive screening test and lower quality of life than the PD group, and significantly higher levels of IL‐1β, TNF‐α, and IL‐4, compared to the patients with NPD. Furthermore, IL‐1β, TNF‐α, and IL‐4 did mediate the relationship between psychological distress and cognitive function in breast cancer survivors. To be specific, the higher level of psychological distress predicted a higher level of cognitive impairment based on the MMSE score, indirectly through the higher level of IL‐1β, TNF‐α, and IL‐4. The mediating role of cytokines suggests the path between psychological distress and CRCI occurs in two steps: (1) psychological distress attributes to increased IL‐1β, TNF‐α, and IL‐4; (2) IL‐1β, TNF‐α, and IL‐4, are associated with CRCI. Here we proposed important ideas of IL‐1β, TNF‐α, and IL‐4; as the underlying CRCI pathway for coping with persistent psychological distress, as well as the potential evidence of psychological intervention to relieve cognitive impairment.

The higher level of psychological distress predicted a higher level of cognitive impairment based on the MMSE score in our study, which further supports the relationship between psychological distress and CRCI.^34^ Self‐reported symptoms of psychological distress can be manifested as anxiety, depression, fear, and nervousness. Chelsea Nicol et al. found that the subjective cognitive function (SCF) including perceived cognitive impairment and perceived cognitive ability of brain tumor survivors were related to pain, emotional disorder, and adverse effects induced by anti‐tumor treatment. Although there were multiple factors affecting the SCF of brain tumors, anxiety was identified to have an independent correlation with SCF.^35^ A systematic review reported that the distress, stress, and loneliness of breast cancer survivors were significantly correlated with the cognitive function of self‐report, which indicated that more psychological distress was associated with more cognitive impairment among breast cancer survivors.^36^ Psychological distress is a repressed emotion that leads to decreased neural activity, and then affects verbal memory and emotional picture accuracy in breast cancer patients.^37^ Therefore, exploring effective psychological intervention to alleviate psychological distress may reverse the symptoms related to CRCI in breast cancer survivors.

Our results found that breast cancer survivors with psychological distress showed significantly higher levels of IL‐1β, TNF‐α, and IL‐4. Previous research also detected the relationship between psychological disorders and changes in cytokines. According to Jones and colleagues, the severe stressor (stress‐enhanced fear learning) increases IL‐1β immunoreactivity and mRNA expression in the dentate gyrus of the dorsal hippocampus (DH), leading to posttraumatic stress disorder (PTSD) in mice, and the treatment of PTSD reduces IL‐1 expression in the DH region as a result of severe stress.^38^ Similarly, breast cancer patients are under the chronic psychological pressure of fear of cancer recurrence and the shortened life length they have to face for a long time, which is likely to lead to changes in neuroinflammation and immune signals, such as cytokine levels, and further aggravate the occurrence of CRCI in breast cancer patients. Recent research suggested that moderate and high levels of TNF‐α strengthen the relationship between perceived social support and psychological distress, which means that appropriate TNF‐α may be the protective cytokine for psychological distress.^39^ This result is not consistent with the present study that higher TNF‐α levels in patients with psychological distress compared with non‐psychological distress, which may be associated with the time after completion of chemotherapy or the presence of other factors that interfere with TNF‐α levels. It has been reported by Linda Witek‐Janusek and colleagues that having the breast biopsied for cancer diagnosis is an emotional experience marked by increased perceived stress, anxiety, and mood disturbances with the increase in IL‐4 production.^40^ Psychological distress is common and almost accompanied by the whole process of cancer, and its relationship with cytokines deserved further study though the indirect effect size of psychological distress on cognitive function through IL‐1β, TNF‐α, and IL‐4 is limited. However, cytokine levels can be altered by infection and cancer treatment, and an extended follow‐up is required to assess the dynamic course of IL‐1β, TNF‐α, and IL‐4 with psychological distress.

Our findings indicated that breast cancer patients with psychological distress had inferior QoL compared with the patients without psychological distress. QoL is influenced by physical, mental, and social/family support.^41^ Aside from the treatment's side effects, psychological disorders including depression, fatigue, and psychological distress can reduce the well‐being of patients. Statistics indicate that less than a third of Eastern Mediterranean breast cancer patients have a good quality of life, an integrated and multidimensional educational program is necessary to improve the quality of life of breast cancer patients.^42^ There is evidence that breast cancer patients who have stronger social support have a higher resilience to stress and better quality of life.^43^, ^44^ This study explored how psychological distress impacts the quality of life in breast cancer patients, providing evidence that psychological intervention can enhance their happiness in life.

There are some limitations to our discussion. This study, conducted in a single center, had a small sample size. The study cannot be guaranteed to be representative due to the small sample size. These results will need to be validated with a larger sample size in the future. MMSE was a cognitive screening test and may not be very sensitive to assess the dimensions of cognition. The measurement of patients' psychological distress is easily affected by the instantaneous emotional state and needs to be evaluated repeatedly to verify the accuracy. In addition, no long‐term dynamic changes in cognitive function and cytokine levels over time were observed.

## 5 CONCLUSION

Our study suggests that breast cancer patients with psychological distress have worse cognitive functioning based on MMSE and lower quality of life, with higher levels of IL‐1β, TNF‐α, and IL‐4. This study demonstrated that psychological distress contributed to CRCI by influencing cytokines level, further explores the psychological and physiological mechanisms of CRCI, and provides evidence for effective psychological intervention in CRCI in breast cancer survivors.

## AUTHOR CONTRIBUTIONS

**Lulian Pang:** Conceptualization (equal); data curation (equal); formal analysis (equal); methodology (equal); writing – original draft (equal); writing – review and editing (equal). **Wen Li:** Conceptualization (equal); data curation (equal); formal analysis (equal); methodology (equal); writing – original draft (equal); writing – review and editing (equal). **Senbang Yao:** Resources (equal). **Yanyan Jing:** Resources (equal). **Xiangxiang Yin:** Resources (equal). **Huaidong Cheng:** Conceptualization (lead); funding acquisition (lead); methodology (equal).

## FUNDING INFORMATION

A grant from the National Natural Science Foundation of China (No. 81872504) supported this study.

## CONFLICT OF INTEREST STATEMENT

There are no competing interests among the authors.

## ETHICS APPROVAL

The Biomedical Ethics Committee of Anhui Medical University approved this study (Project#20131028). Participants of this study signed informed consent before the study data was collected. The study followed the Declaration of Helsinki.

## CONSENT FOR PUBLICATION

Publishing was agreed upon by all authors.

## Data Availability

All data coming from participants are true and reliable, all questionnaire scales were professionally recognized and generally applicable in China, which is available online.

## References

[1] Sung H , Ferlay J , Siegel RL , et al. Global cancer statistics 2020: GLOBOCAN estimates of incidence and mortality worldwide for 36 cancers in 185 countries. CA Cancer J Clin. 2021;71:209‐249. 33538338 10.3322/caac.21660

[2] Cao W , Chen HD , Yu YW , Li N , Chen WQ . Changing profiles of cancer burden worldwide and in China: a secondary analysis of the global cancer statistics 2020. Chin Med J (Engl). 2021;134:783‐791. 33734139 10.1097/CM9.0000000000001474 PMC8104205

[3] DeSantis CE , Ma J , Gaudet MM , et al. Breast cancer statistics, 2019. CA Cancer J Clin. 2019;69:438‐451. 31577379 10.3322/caac.21583

[4] Joensuu H , Kellokumpu‐Lehtinen P , Huovinen R , et al. Adjuvant capecitabine for early breast cancer: 15‐year overall survival results from a randomized trial. J Clin Oncol. 2022;40:1051‐1058. 35020465 10.1200/JCO.21.02054 PMC8966968

[5] Zhou W , Tian W , Xia J , et al. Alterations in degree centrality and cognitive function in breast cancer patients after chemotherapy. Brain Imaging Behav. 2022;16:2248‐2257. 35689165 10.1007/s11682-022-00695-w

[6] Whittaker A , George R , O'Malley L . Prevalence of cognitive impairment following chemotherapy treatment for breast cancer: a systematic review and meta‐analysis. Sci Rep. 2022;12:2135. 35136066 10.1038/s41598-022-05682-1 PMC8826852

[7] Cerulla Torrente N , Navarro Pastor J , de la Osa Chaparro N . Systematic review of cognitive sequelae of non‐central nervous system cancer and cancer therapy. J Cancer Surviv. 2020;14:464‐482. 32146576 10.1007/s11764-020-00870-2

[8] Maeir T , Nahum M , Makranz C , Tsabari S , Peretz T , Gilboa Y . Predictors of quality of life among adults with self‐reported cancer related cognitive impairment. Disabil Rehabil. 2022;45:1056‐1062. 35297702 10.1080/09638288.2022.2050954

[9] Chapman B , Derakshan N , Grunfeld E . Exploring primary breast cancer survivors' self‐management of sustained cancer‐related cognitive impairment in the workplace. Psychooncology. 2022;31:606‐613. 34699652 10.1002/pon.5844

[10] de Ruiter M , Reneman L , Boogerd W , et al. Cerebral hyporesponsiveness and cognitive impairment 10 years after chemotherapy for breast cancer. Hum Brain Mapp. 2011;32:1206‐1219. 20669165 10.1002/hbm.21102 PMC6869999

[11] Onzi G , D'Agustini N , Garcia S , et al. Chemobrain in breast cancer: mechanisms, clinical manifestations, and potential interventions. Drug Saf. 2022;45:601‐621. 35606623 10.1007/s40264-022-01182-3

[12] de la Hoz‐Camacho R , Rivera‐Lazarín A , Vázquez‐Guillen J , et al. Cyclophosphamide and epirubicin induce high apoptosis in microglia cells while epirubicin provokes DNA damage and microglial activation at sub‐lethal concentrations. EXCLI J. 2022;21:197‐212. 35145370 10.17179/excli2021-4160 PMC8822306

[13] Duran‐Gomez N , Lopez‐Jurado CF , Nadal‐Delgado M , Perez‐Civantos D , Guerrero‐Martin J , Caceres MC . Chemotherapy‐related cognitive impairment in patients with breast cancer based on functional assessment and NIRS analysis. J Clin Med. 2022;11:2363. 35566489 10.3390/jcm11092363 PMC9100963

[14] Tyagi K , Masoom M , Majid H , et al. Role of cytokines in chemotherapy related cognitive impairment of breast cancer patients: a systematic review. Curr Rev Clin Exp Pharmacol. 2022;18(2):110‐119. 10.2174/2772432817666220304212456 35249524

[15] Országhová Z , Mego M , Chovanec M . Long‐term cognitive dysfunction in cancer survivors. Front Mol Biosci. 2021;8:770413. 34970595 10.3389/fmolb.2021.770413 PMC8713760

[16] Kaiser J , Dietrich J , Amiri M , et al. Cognitive performance and psychological distress in breast cancer patients at disease onset. Front Psychol. 2019;10:2584. 31803117 10.3389/fpsyg.2019.02584 PMC6873390

[17] Dijkshoorn A , van Stralen H , Sloots M , Schagen S , Visser‐Meily J , Schepers V . Prevalence of cognitive impairment and change in patients with breast cancer: a systematic review of longitudinal studies. Psychooncology. 2021;30:635‐648. 33533166 10.1002/pon.5623 PMC8248098

[18] Patel S , Wong A , Wong F , et al. Inflammatory biomarkers, comorbidity, and neurocognition in women with newly diagnosed breast cancer. J Natl Cancer Inst. 2015;107:djv131. 26101331 10.1093/jnci/djv131 PMC4609551

[19] Eggen A , Richard N , Bosma I , et al. Factors associated with cognitive impairment and cognitive concerns in patients with metastatic non‐small cell lung cancer. Neurooncology Pract. 2022;9:50‐58. 10.1093/nop/npab056 PMC8789294 35087675

[20] Yang Y , Hendrix C . Cancer‐related cognitive impairment in breast cancer patients: influences of psychological variables. Asia Pac J Oncol Nurs. 2018;5:296‐306. 29963592 10.4103/apjon.apjon_16_18 PMC5996591

[21] Areklett E , Fagereng E , Bruheim K , Andersson S , Lindemann K . Self‐reported cognitive impairment in cervical cancer survivors: a cross‐sectional study. Psychooncology. 2022;31:298‐305. 34516040 10.1002/pon.5818

[22] Chen V , Lin C , Hsiao H , et al. Effects of cancer, chemotherapy, and cytokines on subjective and objective cognitive functioning among patients with breast cancer. Cancer. 2021;13:2576. 10.3390/cancers13112576 PMC8197334 34073990

[23] Meyers C , Albitar M , Estey E . Cognitive impairment, fatigue, and cytokine levels in patients with acute myelogenous leukemia or myelodysplastic syndrome. Cancer. 2005;104:788‐793. 15973668 10.1002/cncr.21234

[24] Chan A , Cheng I , Wang C , et al. Cognitive impairment in adolescent and young adult cancer patients: pre‐treatment findings of a longitudinal study. Cancer Med. 2022;12(4):4821‐4831. 36221816 10.1002/cam4.5295 PMC9972136

[25] Pagoni P , Korologou‐Linden R , Howe L , et al. Causal effects of circulating cytokine concentrations on risk of Alzheimer's disease and cognitive function. Brain Behav Immun. 2022;104:54‐64. 35580794 10.1016/j.bbi.2022.05.006 PMC10391322

[26] Yu S , Li W , Tang L , et al. Depression in breast cancer patients: immunopathogenesis and immunotherapy. Cancer Lett. 2022;536:215648. 35307487 10.1016/j.canlet.2022.215648

[27] Holland J , Andersen B , Breitbart W , et al. Distress management. J Natl Compr Canc Netw. 2013;11:190‐209. 23411386 10.6004/jnccn.2013.0027

[28] Donovan K , Grassi L , McGinty H , Jacobsen P . Validation of the distress thermometer worldwide: state of the science. Psychooncology. 2014;23:241‐250. 25160838 10.1002/pon.3430

[29] Folstein M , Folstein S , McHugh P . "Mini‐mental state". A practical method for grading the cognitive state of patients for the clinician. J Psychiatr Res. 1975;12:189‐198. 1202204 10.1016/0022-3956(75)90026-6

[30] Smith G , Della Sala S , Logie R , Maylor E . Prospective and retrospective memory in normal ageing and dementia: a questionnaire study. Memory (Hove, England). 2000;8:311‐321. 11045239 10.1080/09658210050117735

[31] Cella D , Tulsky D , Gray G , et al. The functional assessment of cancer therapy scale: development and validation of the general measure. J Clin Oncol. 1993;11:570‐579. 8445433 10.1200/JCO.1993.11.3.570

[32] Faul F , Erdfelder E , Buchner A , Lang A . Statistical power analyses using G*power 3.1: tests for correlation and regression analyses. Behav Res Methods. 2009;41:1149‐1160. 19897823 10.3758/BRM.41.4.1149

[33] Igartua J , Hayes A . Mediation, moderation, and conditional process analysis: concepts, computations, and some common confusions. Span J Psychol. 2021;24:e49. 35923144 10.1017/SJP.2021.46

[34] Menning S , de Ruiter M , Veltman D , et al. Multimodal MRI and cognitive function in patients with breast cancer prior to adjuvant treatment–the role of fatigue. Neuroimage Clin. 2015;7:547‐554. 25844311 10.1016/j.nicl.2015.02.005 PMC4375788

[35] Nicol C , Ownsworth T , Cubis L , Nguyen W , Foote M , Pinkham M . Subjective cognitive functioning and associations with psychological distress in adult brain tumour survivors. J Cancer Surviv. 2019;13:653‐662. 31313128 10.1007/s11764-019-00784-8

[36] Henneghan A . Modifiable factors and cognitive dysfunction in breast cancer survivors: a mixed‐method systematic review. Support Care Cancer. 2016;24:481‐497. 26416490 10.1007/s00520-015-2927-y

[37] Wirkner J , Weymar M , Löw A , et al. Cognitive functioning and emotion processing in breast cancer survivors and controls: an ERP pilot study. Psychophysiology. 2017;54:1209‐1222. 28432781 10.1111/psyp.12874

[38] Jones M , Lebonville C , Barrus D , Lysle D . The role of brain interleukin‐1 in stress‐enhanced fear learning. Neuropsychopharmacology. 2015;40:1289‐1296. 25430780 10.1038/npp.2014.317 PMC4367475

[39] Perez‐Tejada J , Labaka A , Pascual‐Sagastizabal E , Garmendia L , Iruretagoyena A , Arregi A . Predictors of psychological distress in breast cancer survivors: a biopsychosocial approach. Eur J Cancer Care (Engl). 2019;28:e13166. 31571327 10.1111/ecc.13166

[40] Witek‐Janusek L , Gabram S , Mathews H . Psychologic stress, reduced NK cell activity, and cytokine dysregulation in women experiencing diagnostic breast biopsy. Psychoneuroendocrinology. 2007;32:22‐35. 17092654 10.1016/j.psyneuen.2006.09.011 PMC3937868

[41] Hamer J , McDonald R , Zhang L , et al. Quality of life (QOL) and symptom burden (SB) in patients with breast cancer. Support Care Cancer. 2017;25:409‐419. 27696078 10.1007/s00520-016-3417-6

[42] Hashemi S , Balouchi A , Al‐Mawali A , et al. Health‐related quality of life of breast cancer patients in the eastern Mediterranean region: a systematic review and meta‐analysis. Breast Cancer Res Treat. 2019;174:585‐596. 30632022 10.1007/s10549-019-05131-0

[43] Samami E , Elyasi F , Mousavinasab S , Shojaee L , Zaboli E , Shahhosseini Z . The effect of a supportive program on coping strategies and stress in women diagnosed with breast cancer: a randomized controlled clinical trial. Nurs Open. 2021;8:1157‐1167. 34482657 10.1002/nop2.728 PMC8046153

[44] Zhao X , Tong S , Yang Y . The correlation between quality of life and positive psychological resources in cancer patients: a meta‐analysis. Front Psychol. 2022;13:883157. 35783766 10.3389/fpsyg.2022.883157 PMC9245894

